# Malonate given at reperfusion prevents post-myocardial infarction heart failure by decreasing ischemia/reperfusion injury

**DOI:** 10.1007/s00395-024-01063-z

**Published:** 2024-06-12

**Authors:** Jiro Abe, Ana Vujic, Hiran A. Prag, Michael P. Murphy, Thomas Krieg

**Affiliations:** 1 Department of Medicine, University of Cambridge, Hills Road, Cambridge, CB2 0QQ UK; 2 MRC Mitochondrial Biology Unit, University of Cambridge, Hills Road, Cambridge, CB2 0XY UK

**Keywords:** Malonate, Ischemia/reperfusion injury, Succinate, Mitochondria, Heart failure with reduced ejection fraction, Reactive oxygen species

## Abstract

The mitochondrial metabolite succinate is a key driver of ischemia/reperfusion injury (IRI). Targeting succinate metabolism by inhibiting succinate dehydrogenase (SDH) upon reperfusion using malonate is an effective therapeutic strategy to achieve cardioprotection in the short term (< 24 h reperfusion) in mouse and pig in vivo myocardial infarction (MI) models. We aimed to assess whether inhibiting IRI with malonate given upon reperfusion could prevent post-MI heart failure (HF) assessed after 28 days. Male C57BL/6 J mice were subjected to 30 min left anterior coronary artery (LAD) occlusion, before reperfusion for 28 days. Malonate or without-malonate control was infused as a single dose upon reperfusion. Cardiac function was assessed by echocardiography and fibrosis by Masson’s trichrome staining. Reperfusion without malonate significantly reduced ejection fraction (~ 47%), fractional shortening (~ 23%) and elevated collagen deposition 28 days post-MI. Malonate, administered as a single infusion (16 mg/kg/min for 10 min) upon reperfusion, gave a significant cardioprotective effect, with ejection fraction (~ 60%) and fractional shortening (~ 30%) preserved and less collagen deposition. Using an acidified malonate formulation, to enhance its uptake into cardiomyocytes via the monocarboxylate transporter 1, both 1.6 and 16 mg/kg/min 10 min infusion led to robust long-term cardioprotection with preserved ejection fraction (> 60%) and fractional shortening (~ 30%), as well as significantly less collagen deposition than control hearts. Malonate administration upon reperfusion prevents post-MI HF. Acidification of malonate enables lower doses of malonate to also achieve long-term cardioprotection post-MI. Therefore, the administration of acidified malonate upon reperfusion is a promising therapeutic strategy to prevent IRI and post-MI HF.

## Introduction

**Fig. 1:**
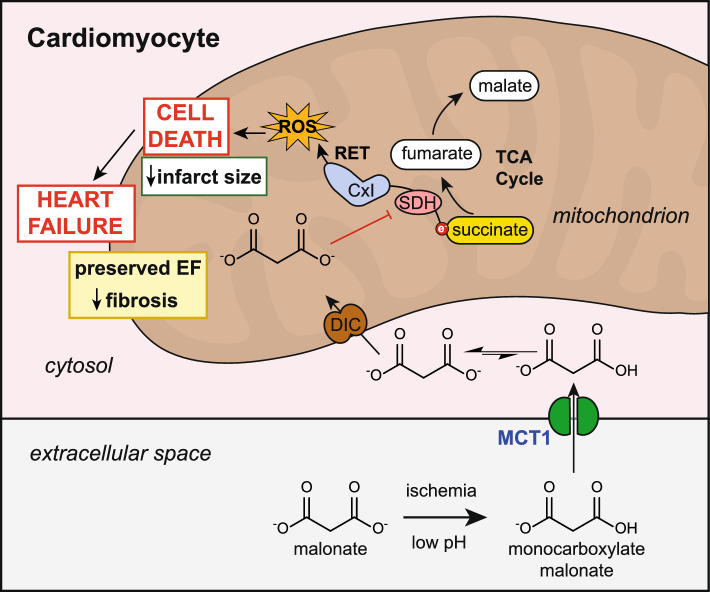
Schematic of malonate inhibition of IRI in MI. Ischemia-accumulated succinate is rapidly oxidized on reperfusion by SDH upon reperfusion. This drives the ROS burst at reperfusion via RET at complex I, which subsequently primes cardiomyocytes for cell death and leads to post-MI HF. Malonate inhibits SDH, slowing down succinate oxidation and ROS production on reperfusion, reducing cell death. Malonate entry into cardiomyocytes can be enhanced by ischemia or low pH, driving the entry of monocarboxylate malonate via MCT1. Whether malonate can prevent post-MI HF by preventing RET-ROS on reperfusion is currently unknown. *MCT1* monocarboxylate transporter 1, *DIC* dicarboxylate carrier, *SDH* succinate dehydrogenase, *CxI* complex I, *RET* reverse electron transport, *ROS* reactive oxygen species, *TCA* tricarboxylic acid cycle, *EF* ejection fraction

There are currently no therapies to prevent ischemia/reperfusion injury (IRI) in myocardial infarction (MI), and thus, it remains an unmet clinical need [13, 15, 23]. Therapies that decrease myocardial damage from IRI may also lower the long-term damage of an MI and thereby reduce the risk of subsequently developing heart failure (HF) [3, 12, 13, 15, 19, 23]. Recently, targeting mitochondria and, in particular, the metabolism of the mitochondrial metabolite succinate has emerged as a promising strategy to prevent IRI. Malonate, a competitive inhibitor of succinate dehydrogenase (SDH), can reduce infarct size when given upon reperfusion in small (8 mg/kg/min over 20 min, starting 5 min before reperfusion in a mouse MI model) [20, 26] and large animal (10 mM intracoronary infusion over 6 min, starting 1 min before reperfusion in a pig MI model) [6, 27] models of acute MI [4, 20, 24, 26, 27]. Malonate is protective in MI by blocking mitochondrial succinate oxidation by SDH; this prevents the burst of reactive oxygen species (ROS) production by reverse electron transport (RET) at mitochondrial complex I, which initiates the damage in IRI [4, 5, 20, 23]. Additionally, malonate has the added benefit that its uptake into tissue is enhanced by ischemia [20]. In ischemia, the acidic pH and lactate accumulation drive malonate uptake into cardiomyocytes through the protonation of malonate to a monocarboxylate that is a substrate for the highly conserved plasma membrane monocarboxylate transporter 1 (MCT1) carrier (Fig. 1) [20, 21]. This mechanism of malonate uptake can also be exploited by administering an acidified malonate formulation, which significantly lowers the dose of malonate required for cardioprotection [20]. While malonate has shown robust protection against IRI in acute MI models, longer term cardiac outcomes of preventing acute IRI with malonate have not been explored [20, 25]. In particular, preventing acute myocardial damage with malonate during MI may have a beneficial effect on the subsequent development of heart failure; however, experimental evidence for this is lacking [20, 25]. Mitochondria are critical targets in IR injury, due to their central role in metabolism and oxygen handling. Therefore, mitochondria are an important target to ameliorate IR injury despite a lack of success in the past [2, 8, 14, 23]. Malonate presents a significant advantage over numerous other mitoprotective strategies, particularly because it targets the initiating cause of damage in IR injury [11, 16, 23, 31]. Other approaches focus on processes downstream of the initiation of damage; therefore, amplification of the damage cascade has already started making it too late for the intervention to be successful [11, 15, 16, 23]. Furthermore, the very rapid uptake of malonate into cardiomyocytes and mitochondria is a significant advantage over other mitoprotective agents which usually rely on slower uptake processes to reach the target [7, 20, 24]. Here, we have determined whether the acute administration of malonate during reperfusion in a murine MI model protected against the chronic organ damage that leads to post-MI heart failure.

**Methods**

### Animals

Procedures were carried out following the UK Animals (Scientific Procedures) Act of 1986 and the University of Cambridge Animal Welfare Policy under project license PP4344323, reviewed by the Animal Welfare Ethical Review Board. Male C57BL/6 J mice aged 10–12 weeks (Charles River Laboratories, UK) were used in this study and maintained on ad libitum access to laboratory chow and water.

### In vivo mouse model of myocardial infarction

The left anterior descending coronary artery (LAD) was occluded to induce MI in an acute open chest, in situ mouse model as described previously [19]. Mice were randomly assigned to treatment groups and operators blinded to treatments. Mice were premedicated with buprenorphine (0.05 mg/kg SC) 10 min prior to surgery, anaesthesia induced with ketamine (100 mg/kg IP) and xylazine (10 mg/kg IP) and maintained on isofluorane in 20% O_2_. Ventilation frequency was maintained at 120–150 breaths/min with tidal volume between 100 and 240 µl. All animals were subjected to 30 min occlusion by placing a suture around the prominent branch of the LAD, through a small plastic tube which was pressed against the heart to induce ischemia followed by 28 day reperfusion. Mice received either 100 µl intravenous saline,160 mg/kg (16 mg/kg/min) disodium malonate (neutral), or 16/160 mg/kg (1.6/16 mg/kg/min) acidified malonate (pH 6) infusions initiated 5 min before the onset of reperfusion for a total of 10 min.

### LC–MS/MS quantification of malonate

Malonate was extracted from tissues and serum and analysed by LC–MS/MS as described in detail previously [20, 22, 24]. 50 µl blood was removed from the animal via the tail vein, left to clot, and centrifuged (2000 × *g*, 10 min, 4 °C) before 5 µl serum extracted in 75 µl extraction buffer containing ^13^C_3_-malonate as an internal standard (IS). 10 mg tissues were homogenized in 250 µl extraction buffer with IS using a Precellys tissue homogenizer (6500 rpm, 15 s × 2; Bertin Instruments, France). Samples were centrifuged (17,000 × *g*, 4 °C, 10 min) and supernatant recentrifuged at the same settings. The resulting supernatant was transferred to MS vials and stored at -70 °C until LC–MS/MS analysis.

### Echocardiography

Echocardiography under anesthesia was performed as recently described [10]. Echo was performed 72 h prior to surgery and 72 h and 28 days after surgery with a Visual sonics Vevo 3100 system (Fujifilm Inc., Japan). The investigator performing the echo analyses was blinded to the intervention status of each mouse.

### Histological staining for fibrosis

After 28 days, the hearts were excised, stored overnight in 10% formalin, and embedded in paraffin before the hearts were sectioned in 10 μm-thick slices throughout the heart and thaw mounted on slides. Masson’s trichrome staining was performed according to the manufacturer’s instructions (Sigma, HT15, USA). Collagen in total area of heart was quantified using ImageJ. The investigator performing histological analysis was blinded to the intervention status of each mouse heart.

### Statistics

Statistical significance was assessed by one- or two-way ANOVA with Dunnett’s post hoc test comparing malonate-treated vs without-malonate control animals. A p value of < 0.05 was considered significant. Statistics were calculated in Prism 10 software (GraphPad Software Inc., USA).

## Results and discussion

The cardioprotective effect of malonate in MI models has usually been assessed after short periods of reperfusion (typically 2 h) by histological measurement of infarct size [18, 20]. While this acute protection is expected to correlate with long-term cardioprotection and function preservation, the infarct at 2 h is not fully developed. Many subsequent factors, including the innate and adaptive immune systems, contribute to the final level of damage to the heart [12, 13, 17]. Therefore, it is important to understand whether malonate inhibition of the RET-ROS burst upon reperfusion after ischemia gives long-term protection to heart function.

**Fig. 2:**
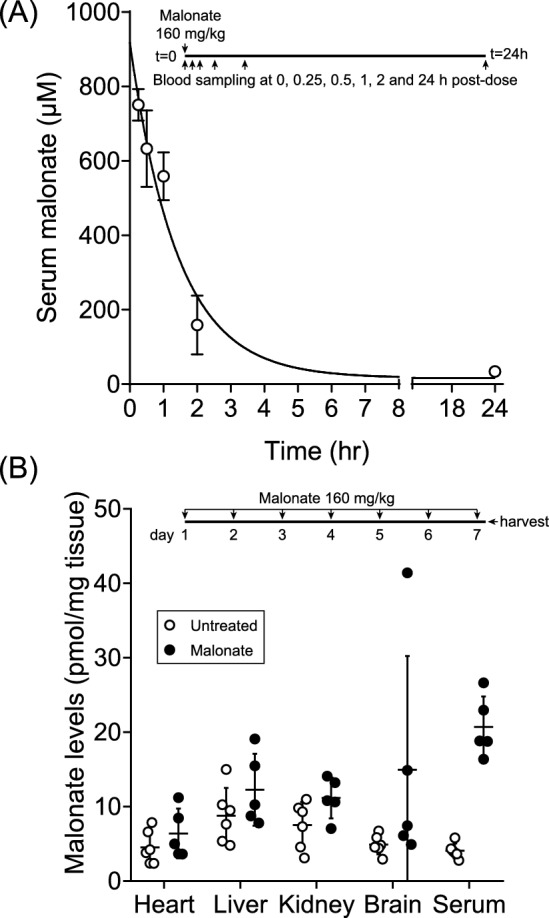
Pharmacokinetics of malonate in vivo. **A** Malonate action is short-lived in vivo. C57BL/6 J mice were IP injected malonate (160 mg/kg as disodium malonate (DSM); 100 µl bolus in neutral pH saline) and serial blood samples taken at 0, 15, 30, 60, 120 min and 24 h after injection. Plasma malonate was assessed by LC–MS/MS. **B** Malonate does not accumulate in tissues. C57BL/6J mice were IP injected daily (160 mg/kg as DSM; 100 µl bolus in neutral pH saline) for 7 days before harvesting tissues 24 h after the last dose and measuring tissue malonate by LC–MS/MS. Data presented as mean ± S.D. from N = 4 biological replicates

First, we assessed the pharmacokinetics of malonate after a single IV dose to understand the persistence of any malonate protective effects beyond its rapid inhibition of ROS production by SDH inhibition in cardiomyocytes upon reperfusion, or whether off-target, longer term pharmacological effects could also be responsible. To do this, we injected malonate IV, at a dose (160 mg/kg bolus) that gives robust protection and measured plasma malonate over time (Fig. 2A). Malonate was rapidly cleared from the blood, with a plasma half-life (t_1/2_) of ~ 0.6 h (Fig. 2A). This suggests that the pharmacological effects of a single IV bolus of malonate are limited to the first few minutes after administration, with limited subsequent effects. To extend this observation, we injected mice daily for 7 days with a single bolus of malonate (160 mg/kg/day) and then measured tissue malonate levels (Fig. 2B). Malonate did not accumulate in any of the tissues assessed (Fig. 2B), further suggesting that acute malonate administration in the MI model only acts to inhibit RET-ROS production upon reperfusion without affecting the later stages of infarct development. Furthermore, mice tolerate large, repeated malonate doses without harm, predicting a good safety profile upon translation of a single bolus of malonate at reperfusion to humans. These experiments suggest that malonate would inhibit RET-ROS and subsequent IRI in MI, with minimal impact on later stages of infarct development.

To assess whether the acute cardioprotection afforded by malonate persists long term, we used a similar protocol to that used previously for acute protection (30 min ischemia by LAD occlusion) [20]; however in this case, cardiac function was assessed by echocardiography and pathological remodelling by Masson’s trichrome staining after 28 days of reperfusion. Malonate was infused at the clinically relevant point of reperfusion, starting 5 min before reperfusion and maintained for 10 min.

**Fig. 3:**
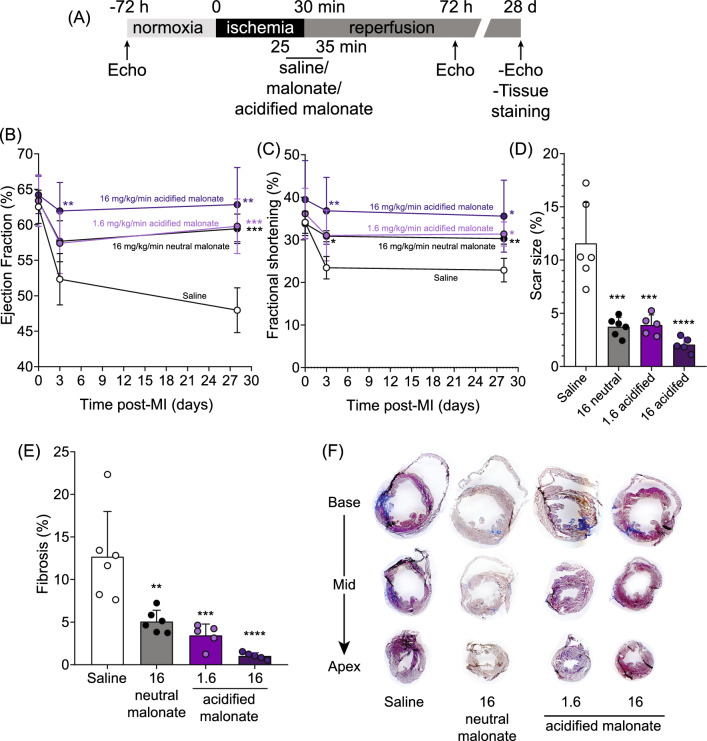
Malonate infusion on reperfusion prevents post-MI HF. C57BL/6 J mice were subjected to 30 min LAD ligation prior to 28 day reperfusion ± 16 mg/kg/min 10 min infusion of malonate (pH 7.4), 1.6 or 16 mg/kg/min 10 min infusion of acidified malonate (pH 6) or saline control, experimental outline depicted in (**A**). Cardiac function was monitored for ejection fraction (**B**) and fractional shortening (**C**) by echocardiography at baseline, 3 and 28 days post-MI. Quantification of scar size as a percentage of LV area (**D**) and fibrosis (**E**) of heart Sections 28 days post-MI and representative stained heart images (**E**). Data presented as mean ± S.D. of N = 5–6 biological replicates. *p < 0.05, **p < 0.01, ***p < 0.001, ****p < 0.0001 versus without malonate-treated animals

Next, we used a dose of neutral malonate (16 mg/kg/min 10 min infusion) known to be cardioprotective when administered upon reperfusion in acute studies. The protection afforded by malonate was evident 28 days post-MI, compared to non-malonate-treated animals (Fig. 3B). Baseline ejection fraction (EF), measured before the induction of ischemia, was no different between the two cohorts of animals (EF ~ 60%); however, while saline-treated animals EF declined over the 28 days post-MI (EF ~ 47%) indicative of heart failure with reduced ejection fraction (HFrEF), neutral malonate-treated animals maintained cardiac function, with end EF values close to baseline. Decreased fractional shortening in control animals over the 28 days post-MI further indicated HFrEF in control animals. In contrast, there was no difference in FS from baseline in the malonate-treated mice (Fig. 3C). Masson’s trichrome staining of 28 day post-MI hearts showed significant collagen deposition in control animals, while the malonate-treated animals had little collagen deposition (Fig. 3D,E). Therefore, malonate inhibition of SDH upon reperfusion ameliorated acute IRI, which consequently prevented the downstream pathological remodelling that leads to HFrEF.

To assess whether the enhanced uptake of protonated malonate via MCT1 uptake could lead to long-term protection at lower malonate doses, we used acidified malonate (pH 6) at 1.6 or 16 mg/kg/min 10 min infusion. Animals treated with either dose upon reperfusion were profoundly cardioprotected, with EF no different from baseline, 28 days post-MI (Fig. 3B). FS was also preserved in acidified malonate-treated animals, whereas saline-treated animals had reduced FS at the end of the study (Fig. 3C). Reperfusion with acidified malonate at either dose significantly reduced collagen deposition in the hearts, and suggested that 1.6 mg/kg/min 10 min infusion of acidified malonate is as effective as 16 mg/kg/min 10 min infusion of neutral malonate (Figs. 3D,E). Therefore, the acidification of malonate enhances its uptake into cardiomyocytes via MCT1, which inhibits RET-ROS and IRI, thereby preventing pathological remodelling and cardiac dysfunction post-MI, at lower doses than neutral malonate.

## Limitations

While the data in this report support the model presented in Fig. 1, that inhibiting succinate-derived RET-ROS with malonate prevents acute IR injury and thereby the subsequent development of HF, greater insights into the pathways and mechanisms of protection from HF are required in future work. As malonate is a competitive inhibitor of SDH, a key enzyme in the Krebs cycle, significant exposure over prolonged periods may contribute to mitochondrial dysfunction, as similarly seen with non-reversible SDH inhibitors or genetic mutations affecting SDH [9, 29, 30]. There remain little data on the long-term administration of malonate and how it may impact mitochondria and metabolism, though its rapid excretion may limit accumulation in tissues. However, as was the case in this study, the use of a single malonate dose upon reperfusion is likely to have minimal pathological effects, with succinate levels being able to outcompete malonate once it is sufficiently redistributed. While acidifying malonate may also limit its off-target effects by reducing the total dose/exposure of the organs to malonate, together with the rapid buffering of the blood reducing uptake, data for this are currently lacking. However, as the acidified malonate is being administered intravenously, exposure of malonate at a lowered pH may be enhanced for the heart compared to other tissues. Other organs that can transport malonate in an MCT1-independent manner, such as the kidneys, would be exposed to a lower dose of malonate when using acidified malonate; however, these comparisons are required to fully understand the off-target effects.

Furthermore, all mice were male; thus, whether similar strong cardioprotective effects are evident in females is also of interest. Also, whether malonate impacts other pathways which could lead to the prevention of HF is currently unknown. While dimethyl malonate (DMM) has been shown to have a potential role in cardiomyocyte proliferation to counteract HF, the mechanisms responsible are likely to be quite distinct from the effect of a single malonate infusion on reperfusion that we have shown here [1]. Bae et al. used a permanent ligation MI model without reperfusion and repeated dosing of DMM. As DMM also showed a lack of acute protection either with repeat administration or a single infusion upon reperfusion [24], this further indicates different mechanisms at play leading to protection of the heart.

## Conclusion

The current standard of care for MI has improved acute MI mortality; however, the subsequent development of heart failure caused by IRI remains a significant unmet clinical need [3]. We have now shown that the acute administration of a single infusion of malonate upon reperfusion leads to long-lasting cardioprotection and prevention of HFrEF development 28 days post-MI. Furthermore, enhancing cardiac malonate uptake through acidification can achieve similar robust, long-term cardioprotection at far lower malonate doses. This work suggests that administering acidified malonate upon reperfusion has significant translational potential in preventing the development of HF post-MI. Furthermore, while this manuscript was in revision, another study showed consistent results to those presented here, in that malonate infusion on reperfusion led to sustained cardiac function 28 days after reperfusion [28]. Whether the prevention of post-MI HF is solely due to a reduction in infarct size or other acute effects of malonate are also responsible requires further investigation.

## References

[1] Bae J, Salamon RJ, Brandt EB, Paltzer WG, Zhang Z, Britt EC, Hacker TA, Fan J, Mahmoud AI (2021) Malonate promotes adult cardiomyocyte proliferation and heart regeneration. Circulation 143:1973–1986. 10.1161/CIRCULATIONAHA.120.049952 33666092 10.1161/CIRCULATIONAHA.120.049952 PMC8131241

[2] Bøtker HE, Cabrera-Fuentes HA, Ruiz-Meana M, Heusch G, Ovize M (2020) Translational issues for mitoprotective agents as adjunct to reperfusion therapy in patients with ST-segment elevation myocardial infarction. J Cell Mol Med 24:2717–2729. 10.1111/jcmm.14953 31967733 10.1111/jcmm.14953 PMC7077531

[3] Bøtker HE, Hausenloy D, Andreadou I, Antonucci S, Boengler K, Davidson SM, Deshwal S, Devaux Y, Di Lisa F, Di Sante M, Efentakis P, Femminò S, García-Dorado D, Giricz Z, Ibanez B, Iliodromitis E, Kaludercic N, Kleinbongard P, Neuhäuser M, Ovize M, Pagliaro P, Rahbek-Schmidt M, Ruiz-Meana M, Schlüter K-D, Schulz R, Skyschally A, Wilder C, Yellon DM, Ferdinandy P, Heusch G (2018) Practical guidelines for rigor and reproducibility in preclinical and clinical studies on cardioprotection. Basic Res Cardiol 113:39. 10.1007/s00395-018-0696-8 30120595 10.1007/s00395-018-0696-8 PMC6105267

[4] Chouchani ET, Pell VR, Gaude E, Aksentijević D, Sundier SY, Robb EL, Logan A, Nadtochiy SM, Ord ENJ, Smith AC, Eyassu F, Shirley R, Hu C, Dare AJ, James AM, Rogatti S, Hartley RC, Eaton S, Costa ASH, Brookes PS, Davidson SM, Duchen MR, Saeb-Parsy K, Shattock MJ, Robinson AJ, Work LM, Frezza C, Krieg T, Murphy MP (2014) Ischaemic accumulation of succinate controls reperfusion injury through mitochondrial ROS. Nature 515:431–435. 10.1038/nature13909 25383517 10.1038/nature13909 PMC4255242

[5] Chouchani ET, Pell VR, James AM, Work LM, Saeb-Parsy K, Frezza C, Krieg T, Murphy MP (2016) A unifying mechanism for mitochondrial superoxide production during ischemia-reperfusion injury. Cell Metab 23:254–263. 10.1016/j.cmet.2015.12.009 26777689 10.1016/j.cmet.2015.12.009

[6] Consegal M, Núñez N, Barba I, Benito B, Ruiz-Meana M, Inserte J, Ferreira-González I, Rodríguez-Sinovas A (2021) Citric acid cycle metabolites predict infarct size in pigs submitted to transient coronary artery occlusion and treated with succinate dehydrogenase inhibitors or remote ischemic perconditioning. Int J Mol Sci. 10.3390/ijms22084151 33923786 10.3390/ijms22084151 PMC8072915

[7] Cung T-T, Morel O, Cayla G, Rioufol G, Garcia-Dorado D, Angoulvant D, Bonnefoy-Cudraz E, Guérin P, Elbaz M, Delarche N, Coste P, Vanzetto G, Metge M, Aupetit J-F, Jouve B, Motreff P, Tron C, Labeque J-N, Steg PG, Cottin Y, Range G, Clerc J, Claeys MJ, Coussement P, Prunier F, Moulin F, Roth O, Belle L, Dubois P, Barragan P, Gilard M, Piot C, Colin P, De Poli F, Morice M-C, Ider O, Dubois-Randé J-L, Unterseeh T, Le Breton H, Béard T, Blanchard D, Grollier G, Malquarti V, Staat P, Sudre A, Elmer E, Hansson MJ, Bergerot C, Boussaha I, Jossan C, Derumeaux G, Mewton N, Ovize M (2015) Cyclosporine before PCI in patients with acute myocardial infarction. N Engl J Med 373:1021–1031. 10.1056/NEJMoa1505489 26321103 10.1056/NEJMoa1505489

[8] Davidson SM, Ferdinandy P, Andreadou I, Bøtker HE, Heusch G, Ibáñez B, Ovize M, Schulz R, Yellon DM, Hausenloy DJ, Garcia-Dorado D (2019) Multitarget strategies to reduce myocardial ischemia/reperfusion injury. J Am Coll Cardiol 73:89–99. 10.1016/j.jacc.2018.09.086 30621955 10.1016/j.jacc.2018.09.086

[9] Gottlieb E, Tomlinson IPM (2005) Mitochondrial tumour suppressors: a genetic and biochemical update. Nat Rev Cancer 5:857–866. 10.1038/nrc1737 16327764 10.1038/nrc1737

[10] Grune J, Ritter D, Kräker K, Pappritz K, Beyhoff N, Schütte T, Ott C, John C, van Linthout S, Tschöpe C, Dechend R, Muller DN, Haase N, Grune T, Kintscher U, Kuebler WM (2019) Accurate assessment of LV function using the first automated 2D-border detection algorithm for small animals - evaluation and application to models of LV dysfunction. Cardiovasc Ultrasound 17:7. 10.1186/s12947-019-0156-0 31010431 10.1186/s12947-019-0156-0 PMC6477743

[11] Hernandez-Resendiz S, Prakash A, Loo SJ, Semenzato M, Chinda K, Crespo-Avilan GE, Dam LC, Lu S, Scorrano L, Hausenloy DJ (2023) Targeting mitochondrial shape: at the heart of cardioprotection. Basic Res Cardiol 118:49. 10.1007/s00395-023-01019-9 37955687 10.1007/s00395-023-01019-9 PMC10643419

[12] Heusch G (2017) Critical issues for the translation of cardioprotection. Circ Res 120:1477–1486. 10.1161/CIRCRESAHA.117.310820 28450365 10.1161/CIRCRESAHA.117.310820

[13] Heusch G (2020) Myocardial ischaemia–reperfusion injury and cardioprotection in perspective. Nat Rev Cardiol 17:773–789. 10.1038/s41569-020-0403-y 32620851 10.1038/s41569-020-0403-y

[14] Heusch G (2024) Myocardial ischemia/reperfusion: translational pathophysiology of ischemic heart disease. Med 5:10–31. 10.1016/j.medj.2023.12.007 38218174 10.1016/j.medj.2023.12.007

[15] Heusch G, Andreadou I, Bell R, Bertero E, Botker H-E, Davidson SM, Downey J, Eaton P, Ferdinandy P, Gersh BJ, Giacca M, Hausenloy DJ, Ibanez B, Krieg T, Maack C, Schulz R, Sellke F, Shah AM, Thiele H, Yellon DM, Di Lisa F (2023) Health position paper and redox perspectives on reactive oxygen species as signals and targets of cardioprotection. Redox Biol 67:102894. 10.1016/j.redox.2023.102894 37839355 10.1016/j.redox.2023.102894 PMC10590874

[16] Kleinbongard P (2023) Perspective: mitochondrial STAT3 in cardioprotection. Basic Res Cardiol 118:32. 10.1007/s00395-023-01003-3 37620559 10.1007/s00395-023-01003-3 PMC10449977

[17] Kleinbongard P, Bøtker HE, Ovize M, Hausenloy DJ, Heusch G (2020) Co-morbidities and co-medications as confounders of cardioprotection—does it matter in the clinical setting? Br J Pharmacol. 10.1111/bph.14839 31430831 10.1111/bph.14839 PMC7680006

[18] Lecour S, Andreadou I, Bøtker HE, Davidson SM, Heusch G, Ruiz-Meana M, Schulz R, Zuurbier CJ, Ferdinandy P, Hausenloy DJ, Adamovski P, Andreadou I, Batirel S, Barteková M, Bertrand L, Beauloye C, Biedermann D, Borutaite V, Bøtker HE, Chlopicki S, Dambrova M, Davidson S, Devaux Y, Di Lisa F, Djuric D, Erlinge D, Falcao-Pires I, Ferdinandy P, Galatou E, Garcia-Sosa A, Girao H, Giricz Z, Gyongyosi M, Hausenloy DJ, Healy D, Heusch G, Jakovljevic V, Jovanic J, Kararigas G, Kerkal R, Kolar F, Kwak B, Leszek P, Liepinsh E, Lonborg J, Longnus S, Marinovic J, Muntean DM, Nezic L, Ovize M, Pagliaro P, Da Costa Gomes CP, Pernow J, Persidis A, Pischke SE, Podesser B, Potočnjak I, Prunier F, Ravingerova T, Ruiz-Meana M, Serban A, Slagsvold K, Schulz R, van Royen N, Turan B, Vendelin M, Walsh S, Zidar N, Zuurbier C, Yellon D (2021) IMproving preclinical assessment of cardioprotective therapies (IMPACT) criteria: guidelines of the EU-CARDIOPROTECTION COST Action. Basic Res Cardiol 116:52. 10.1007/s00395-021-00893-5 34515837 10.1007/s00395-021-00893-5 PMC8437922

[19] Methner C, Chouchani ET, Buonincontri G, Pell VR, Sawiak SJ, Murphy MP, Krieg T (2014) Mitochondria selective S -nitrosation by mitochondria-targeted S -nitrosothiol protects against post-infarct heart failure in mouse hearts. Eur J Heart Fail 16:712–717. 10.1002/ejhf.100 24891297 10.1002/ejhf.100 PMC4231226

[20] Prag HA, Aksentijevic D, Dannhorn A, Giles AV, Mulvey JF, Sauchanka O, Du L, Bates G, Reinhold J, Kula-Alwar D, Xu Z, Pellerin L, Goodwin RJA, Murphy MP, Krieg T (2022) Ischemia-selective cardioprotection by malonate for ischemia/reperfusion injury. Circ Res 131:528–541. 10.1161/CIRCRESAHA.121.320717 35959683 10.1161/CIRCRESAHA.121.320717 PMC9426742

[21] Prag HA, Gruszczyk AV, Huang MM, Beach TE, Young T, Tronci L, Nikitopoulou E, Mulvey JF, Ascione R, Hadjihambi A, Shattock MJ, Pellerin L, Saeb-Parsy K, Frezza C, James AM, Krieg T, Murphy MP, Aksentijević D (2021) Mechanism of succinate efflux upon reperfusion of the ischaemic heart. Cardiovasc Res 117:1188–1201. 10.1093/cvr/cvaa148 32766828 10.1093/cvr/cvaa148 PMC7983001

[22] Prag HA, Kula-Alwar D, Pala L, Caldwell ST, Beach TE, James AM, Saeb-Parsy K, Krieg T, Hartley RC, Murphy MP (2020) Selective delivery of dicarboxylates to mitochondria by conjugation to a lipophilic cation via a cleavable linker. Mol Pharm 17:3526–3540. 10.1021/acs.molpharmaceut.0c00533 32692564 10.1021/acs.molpharmaceut.0c00533 PMC7482397

[23] Prag HA, Murphy MP, Krieg T (2023) Preventing mitochondrial reverse electron transport as a strategy for cardioprotection. Basic Res Cardiol 118:34. 10.1007/s00395-023-01002-4 37639068 10.1007/s00395-023-01002-4 PMC10462584

[24] Prag HA, Pala L, Kula-Alwar D, Mulvey JF, Luping D, Beach TE, Booty LM, Hall AR, Logan A, Sauchanka V, Caldwell ST, Robb EL, James AM, Xu Z, Saeb-Parsy K, Hartley RC, Murphy MP, Krieg T (2022) Ester Prodrugs of malonate with enhanced intracellular delivery protect against cardiac ischemia-reperfusion injury in vivo. Cardiovasc Drugs Ther 36:1–13. 10.1007/s10557-020-07033-6 32648168 10.1007/s10557-020-07033-6 PMC8770414

[25] Schulz R, Heusch G (2022) Targeted mito- and cardioprotection by malonate. Circ Res 131:542–544. 10.1161/CIRCRESAHA.122.321582 36048916 10.1161/CIRCRESAHA.122.321582

[26] Valls-Lacalle L, Barba I, Miró-Casas E, Alburquerque-Béjar JJ, Ruiz-Meana M, Fuertes-Agudo M, Rodríguez-Sinovas A, García-Dorado D (2016) Succinate dehydrogenase inhibition with malonate during reperfusion reduces infarct size by preventing mitochondrial permeability transition. Cardiovasc Res 109:374–384. 10.1093/cvr/cvv279 26705364 10.1093/cvr/cvv279

[27] Valls-Lacalle L, Barba I, Miró-Casas E, Ruiz-Meana M, Rodríguez-Sinovas A, García-Dorado D (2018) Selective inhibition of succinate dehydrogenase in reperfused myocardium with intracoronary malonate reduces infarct size. Sci Rep 8:2442. 10.1038/s41598-018-20866-4 29402957 10.1038/s41598-018-20866-4 PMC5799359

[28] Valls-Lacalle L, Consegal M, Ganse FG, Yáñez-Bisbe L, Pastor J, Ruiz-Meana M, Inserte J, Benito B, Ferreira-González I, Rodríguez-Sinovas A (2024) Long-term protective effects of succinate dehydrogenase inhibition during reperfusion with malonate on post-infarction left ventricular scar and remodeling in mice. Int J Mol Sci 25:4366. 10.3390/ijms25084366 38673951 10.3390/ijms25084366 PMC11050251

[29] Wang X, Zhang X, Cao K, Zeng M, Fu X, Zheng A, Zhang F, Gao F, Zou X, Li H, Li M, Lv W, Xu J, Long J, Zang W, Chen J, Gao F, Ding J, Liu J, Feng Z (2022) Cardiac disruption of SDHAF4-mediated mitochondrial complex II assembly promotes dilated cardiomyopathy. Nat Commun 13:3947. 10.1038/s41467-022-31548-1 35803927 10.1038/s41467-022-31548-1 PMC9270418

[30] Wojtovich AP, Smith CO, Haynes CM, Nehrke KW, Brookes PS (2013) Physiological consequences of complex II inhibition for aging, disease, and the mKATP channel. Biochim Biophys Acta 1827:598–611. 10.1016/j.bbabio.2012.12.007 23291191 10.1016/j.bbabio.2012.12.007 PMC3880126

[31] Yellon DM, Beikoghli Kalkhoran S, Davidson SM (2023) The RISK pathway leading to mitochondria and cardioprotection: how everything started. Basic Res Cardiol 118:22. 10.1007/s00395-023-00992-5 37233787 10.1007/s00395-023-00992-5 PMC10220132

